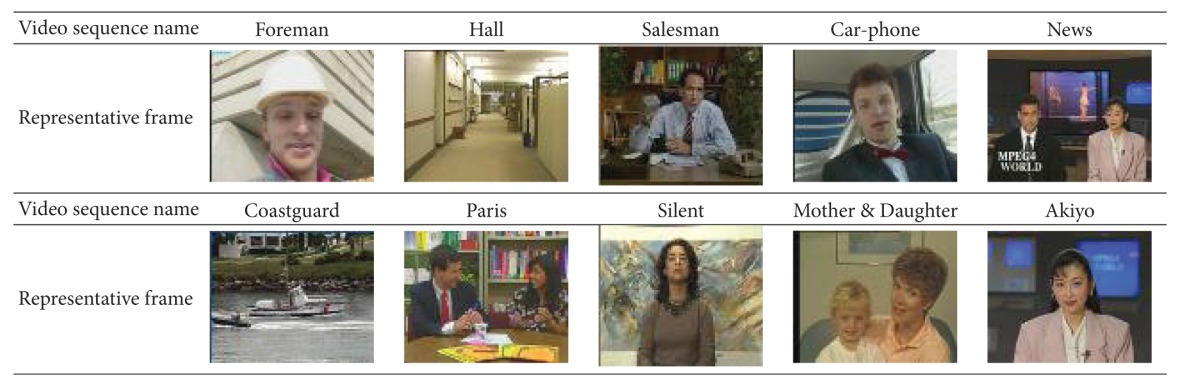# An Efficient Hierarchical Video Coding Scheme Combining Visual Perception Characteristics

**DOI:** 10.1155/2014/727943

**Published:** 2014-05-13

**Authors:** Pengyu Liu, Kebin Jia

**Affiliations:** School of Electronic Information & Control Engineering, Beijing University of Technology, Beijing 100124, China

## Abstract

Different visual perception characteristic saliencies are the key to constitute the low-complexity video coding framework. A hierarchical video coding scheme based on human visual systems (HVS) is proposed in this paper. The proposed scheme uses a joint video coding framework consisting of visual perception analysis layer (VPAL) and video coding layer (VCL). In VPAL, effective visual perception characteristics detection algorithm is proposed to achieve visual region of interest (VROI) based on the correlation between coding information (such as motion vector, prediction mode, etc.) and visual attention. Then, the interest priority setting for VROI according to visual perception characteristics is completed. In VCL, the optional encoding method is developed utilizing the visual interested priority setting results from VPAL. As a result, the proposed scheme achieves information reuse and complementary between visual perception analysis and video coding. Experimental results show that the proposed hierarchical video coding scheme effectively alleviates the contradiction between complexity and accuracy. Compared with H.264/AVC (JM17.0), the proposed scheme reduces 80% video coding time approximately and maintains a good video image quality as well. It improves video coding performance significantly.

## 1. Introduction

Due to the rapid growth of the multimedia service, the video compression becomes essential for reducing the required bandwidth for transmission and storage in many applications. The prospects of video coding technology are broad ranging from national defense, scientific research, education, and medicine to aerospace engineering. However, in the case of limited bandwidth and storage resources, new requirements have been raised for the existing video coding standard, such as higher resolution, higher image quality, and higher frame rate.

In order to achieve low complexity, high quality, and high compression-ratio, the International Telecommunication Union (ITU-T) and the International Organization for Standardization (ISO/IEC) set up a Collaborative Team on Video Coding (JCT-VC) and released the next generation of video coding technology proposal High Efficiency Video Coding (HEVC) [1, 2] in January 2010. HEVC still inherits the hybrid coding framework of H.264/AVC which is launched by ITU-T and ISO/IEC in 2003. HEVC focuses on the study of new video coding techniques to resolve the contradiction between the compression-ratio and coding complexity. More than that HEVC aims at adapting many different types of network transmission and carrying more information processing business [3]. It has become one of the hottest research areas in signal and information processing in the technologies and applications of “real time,” “high compression-ratio,” and “high resolution” [4, 5].

Up to now, many scholars carried out a lot of work on fast video coding algorithm or visual perception analysis, but few of them combine the two kinds of coding technique in a video coding framework to jointly optimize the performance of video coding [6, 7].

Tsapatsoulis et al. [8] detected the region of interest by color, brightness, direction, and complexion, but they ignored the motion visual characteristics [9]. Wang et al. [10] built a model of visual attention to extract region of interest by motion, brightness, face, text, and other visual characteristics. Tang et al. [11, 12] and Lin and Zheng [13] obtained the region of interest by motion and texture. Fang et al. [14, 15] proposed that the region of interest obtains method based on wavelet transform or in the compressed domain. Because the global motion estimation algorithm is too complicated, it is difficult to extract the visual region of interest. The video coding algorithms based on human visual systems (HVS) technology mentioned above focused on the bit resource allocation optimization under limited bit resources. Considering the region of interest, the above video coding methods based on HVS lack computing resource allocation optimization, and the additional computational complexity which was caused by visual perception analysis is neglected also.

On the other hand, Kim et al. [16] reduced the loss of rate-distortion performance under limited computing resource by controlling the motion estimation search points. Saponara et al. [17] adjusted the numbers of reference frames, the prediction mode, and the motion estimation search range according to the sum of difference Sum of Absolute Differences (SAD). Su et al. [18] set the parameters of motion estimation and mode decision to achieve a self-adaptive computational complexity controller. The above computing resource optimizations do not distinguish the various regions according to the saliency of the visual perception. This kind of algorithm ignores the differences of the perception in various video scenes that use the same coding algorithm for all encoding contents in video.

Therefore, there is important theoretical significance in using visual perception principle to optimize the computing resource allocation. The optimization further improves the computational efficiency of the video coding standard. In this paper H.264/AVC (JM17.0) is taken as the experimental platform, where we combine the visual perception analysis and the fast video coding algorithm to make the two respective advantages complementary to each other. The proposed method optimizes computing resource allocation more effectively by using visual perception principle and then proposes an efficient hierarchical video coding algorithm based on visual perception characteristics.

## 2. Visual Perception Characteristics Analysis for VPAL

Rapid and effective visual analysis which can effectively detect the visual region of interest is the key to optimize coding resource. We propose an efficient hierarchical video coding algorithm based on visual perception characteristics.

### 2.1. Temporal Visual Characteristics Analysis and Detection

On the ideal condition, foreground movement brings out a nonzero motion vector which is highly focused by HVS. Because background does not have relative movement, so it brings out a zero motion vector which is lowly focused by HVS. So the motion vector can be regarded as the temporal characteristics of visual perception analysis. While, on the real condition, due to external light change and inherent parameters change such as quantization parameter (QP), motion search strategy, and rate-distortion optimization, nonzero motion vector random noise in background will appear. In addition, the horizontal displacement of camera will bring out global motion vectors. Therefore, it is necessary to develop appropriate motion vector detection to filter motion vector random noise interference and translational motion vector error.

#### 2.1.1. Motion Vector Random Noise Filtering

Motion vector noise filter is put forward based on the following principle.

According to motion continuity and integrity, there is strong correlation of movement characteristics between the current coding block and the corresponding position blocks in the previous frame. We define the motion reference region consisting of the encoded macroblocks having position correlation with the current coding block in the previous frame, signed *C*_*rr*_. If there exists nonzero motion vector $\overset{⃗}{v_{s}}$ in the current coding block, but there is no motion vector in reference region *C*_*rr*_, then considering $\overset{⃗}{v_{s}}$ a motion vector random noise should be filtered.

Therefore, how to define the reference region becomes one of the key factors of motion vector noise filtering results.

**Figure 1:**
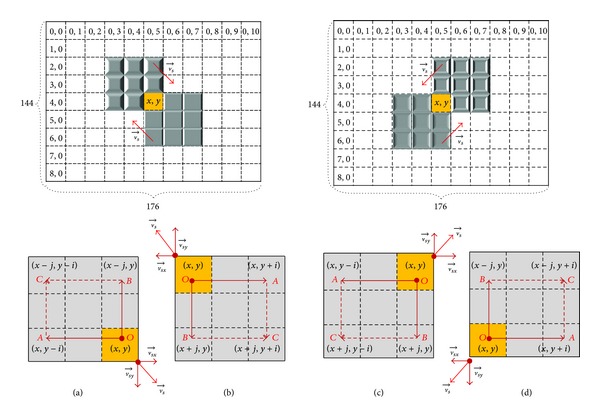
Schematic diagram of motion reference region *C*_*rr*_.

In this paper, taking QCIF format encoded video sequence, for example, *C*_*rr*_ is defined as shown in Figure 1.

In Figure 1, take macroblock *O* as the initial search point, which has the same coordinates (*x*, *y*) as the current coding block, move *i*_*c*_ macroblocks horizontally opposite to $\overset{⃗}{v_{sx}}$, and get macroblock *A*. Then, take macroblock *O* as the initial point again, move *j*_*c*_ macroblocks vertically opposite to $\overset{⃗}{v_{sy}}$, and get macroblock *B*. After that, take macroblocks *A* and *B* as the starting points, make the extension of vertical directions and horizontal directions. respectively, and get macroblock *C*. As a result, obtain a rectangular region surrounded by four macroblocks *O*, *A*, *B*, *C*, namely, motion reference region *C*_*rr*_.

Here, (*x*, *y*) represents the position coordinates of the current coding block. $\overset{⃗}{v_{sx}}$ and $\overset{⃗}{v_{sy}}$ represent the motion vector components in the horizontal directions and vertical directions of $\overset{⃗}{v_{s}}$, respectively. *i* and *j* are defined as

(1) $$\begin{array}{c} i=\mathrm{int}\left(\frac{\left|\overset{⃗}{v_{sx}}\right|}{w_{s}}+1\right),\;\;j=\mathrm{int}\left(\frac{\left|\overset{⃗}{v_{sy}}\right|}{h_{s}}+1\right). \end{array}$$

In formula (1), $\left|\overset{⃗}{v_{sx}}\right|$, $\left|\overset{⃗}{v_{sy}}\right|$ represents the amplitude of $\overset{⃗}{v_{sx}}$ and $\overset{⃗}{v_{sy}}$, and *w*_*s*_, *h*_*s*_ represent the width and height of the current coding block, respectively.

Based on the current the movement direction of $\overset{⃗}{v_{s}}$ (lower right, upper left, upper right, lower left, shown as Figures 1(a)–1(d)), the coordinates of the four macroblocks composition of *C*_*rr*_ can be expressed as {(*x*, *y*), (*x*, *y* − *i*), (*x* − *j*, *y* − *i*), (*x* − *j*, *y*)}, {(*x*, *y*), (*x*, *y* + *i*), (*x* + *j*, *y* + *i*), (*x* + *j*, *y*)}, {(*x*, *y*), (*x* + *j*, *y*), (*x* + *j*, *y* − *i*), (*x*, *y* − *i*)}, {(*x*, *y*), (*x* − *j*, *y*), (*x* − *j*, *y* + *i*), (*x*, *y* + *i*)}, respectively, along the clockwise direction.

If any one of the three macro-blocks *A*, *B*, *C* is not in the encoding frame, which means it is beyond the border of the encoding frame, choose the macroblocks on the boundary as the coordinate points of motion reference region C_rr_.

The method of detect motion vector random noise is defined as

(2) $$\begin{array}{c} M_{1}\left(x,y\right)=\{\begin{array}{cc} 3, & \text{if}\;\;\left|\bar{V_{rr}}\right|=0\\ 2, & \text{else}\;\text{if}\;\left|v_{s}\right|\geq \left|\bar{V_{rr}}\right|\\ 1, & \text{else}\;\text{if}\;\left|v_{s}\right|<\left|\bar{V_{rr}}\right|. \end{array} \end{array}$$

In formula (2), (*x*, *y*) represents the coordinates of the current coding macroblock. $\bar{V_{rr}}$ represents the mean motion vector in *C*_*rr*_.

If $\left|\bar{V_{rr}}\right|=0$, means in *C*_*rr*_, there is no motion vector, *v*_*s*_ is set to 0. *M*_1_(*x*, *y*) = 3, means *v*_*s*_ is caused by motion vector random noise.

If $\left|v_{s}\right|\geq \left|\bar{V_{rr}}\right|$, *M*_1_(*x*, *y*) = 2, means that the current macroblock has more saliency motion characteristics compared with neighbored macroblocks and it belongs to foreground dynamic region.

Otherwise, *M*_1_(*x*, *y*) is set to *M*_2_(*x*, *y*) and then the motion vector is going to be detected whether it is translational or not. The translational motion vector detection can distinguish the macroblock belonging to background region or foreground translational region which has the similar motion characteristics in neighbored macroblocks.

#### 2.1.2. Translational Motion Vector Detection

Consider

(3) $$\begin{array}{c} M_{2}\left(x,y\right)=\{\begin{array}{cc} 1, & \text{if}\;\;{\text{SAD}}_{\left(x,y\right)}=\overset{M,N}{\underset{i=0,j=0}{\sum }}\left|s\left(i,j\right)-c\left(i,j\right)\right|\geq {\bar{S}}_{c}\\ 0, & \text{else}. \end{array} \end{array}$$

In formula (3), (*x*, *y*) represents the coordinates of the current macro-block, *s*(*i*, *j*) represents the pixel of the current macro-block, *c*(*i*, *j*) represents the pixel of the corresponding macroblock in previous frame, and *M* and *N* represent the pixels number in the horizontal or vertical direction of the current macroblock, respectively.

If the value of SAD(*x*, *y*) is larger, the difference of the corresponding macroblocks in neighbored frames is bigger. In this case the current macroblock belongs to foreground translational region in translational background, and then *M*_2_(*x*, *y*) is set to 1. In another case, the current macroblock belongs to background region, and then *M*_2_(*x*, *y*) is set to 0. Because the SAD(*x*, *y*) is calculated in intermode decision and motion estimation, so the translational motion vector detection does not increase more computation, especially fits for limited calculation resources. In order to reduce the detection error, this paper sets up an adaptive dynamic threshold ${\bar{S}}_{c}$ to detect the translational motion vector interference. ${\bar{S}}_{c}$ is the mean SAD of all macroblocks which are considered in background at previous frame:

(4) $$\begin{array}{c} {\bar{S}}_{c}=\frac{\sum_{x,y\in S_{\text{c}}}^{}{\text{SAD}}_{\left(x,y\right)}}{\text{Num}}. \end{array}$$

In formula (4), *S*_*c*_ represents the background region in previous frame, ∑_*x*,*y*∈*S*_*c*__SAD_(*x*,*y*)_ represents the sum of the SAD in *S*_*c*_, and Num represents the summation times.

#### 2.1.3. Temporal Visual Saliency Region Marking

Consider

(5) $$\begin{array}{c} M\left(x,y\right)=\{\begin{array}{cc} 3, & \text{if}\;\;\left|\bar{V_{rr}}\right|=0\\ 2, & \text{else}\;\begin{array}{c} \text{if} \end{array}\;\left|v_{s}\right|\geq \left|\bar{V_{rr}}\right|\\ 1, & \text{else}\;\begin{array}{c} \text{if} \end{array}\;\begin{array}{c} {\text{SAD}}_{\left(x,y\right)}\geq {\bar{S}}_{c} \end{array}\\ 0, & \text{esle}. \end{array} \end{array}$$

In formula (5), *M*(*x*, *y*) = 3 represents motion vector random noise, and after motion vector random noise filtering *M*(*x*, *y*) is set to 0. *M*(*x*, *y*) = 2 represents foreground dynamic region. *M*(*x*, *y*) = 1 represents foreground translational region. *M*(*x*, *y*) = 0 represents background region.

**Figure 2:**
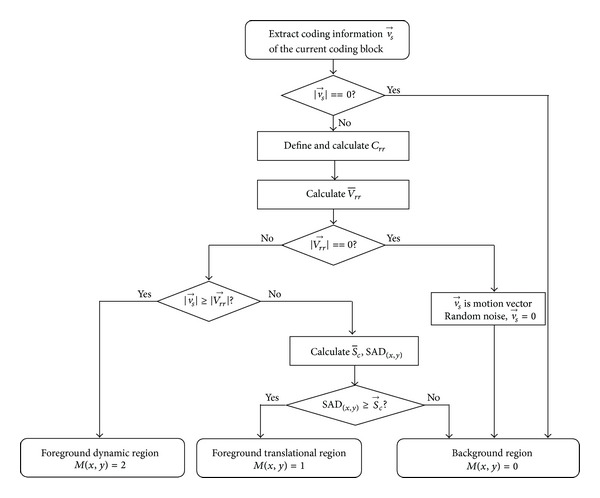
Flowchart of temporal visual saliency region marking.

In this paper, the temporal visual characteristics analysis and detection are realized by preprocessing in two layers; the proposed algorithm flowchart is given in Figure 2.

**Figure 3:**
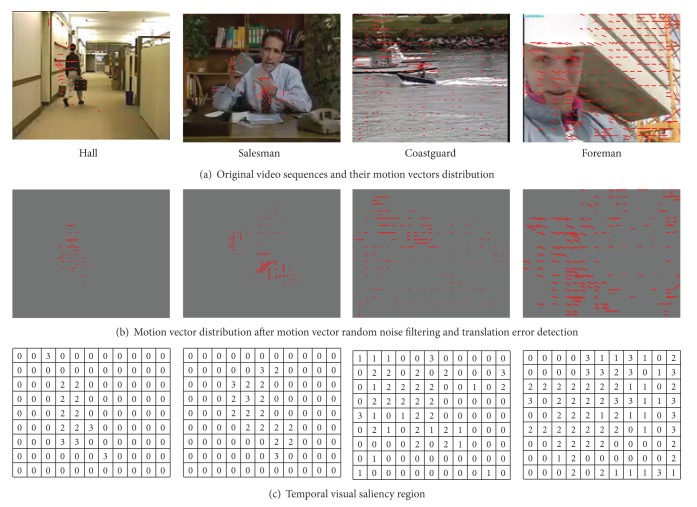
Temporal visual perception characteristics analysis.

The current encoding frame is divided into different temporal visual perception characteristic regions according to $\overset{⃗}{v_{s}}$ and motion vector correlation between neighbored macroblocks.  Figure 3 shows part of the experiment results schematic; taking typical video monitoring sequence (Hall), indoor activity sequence (Salesman), and outdoor sequences (Coastguard, Foreman) including camera panning, for example, it can be found that the proposed method can disperse foreground and background effectively.

### 2.2. Spatial Visual Characteristics Analysis and Detection

Existing research results have proved that mode decision is accordant well to visual attention. The macroblocks choose subblock prediction modes in intraframe or interframe coding with high probability and attended highly by human eyes when spatial visual characteristic varies intensely or abundant image contents include more moving details. The macroblocks have been chosen by macroblock prediction mode in intraframe or interframe coding with high probability and attended lowly by human eyes when spatial visual characteristic varies slowly or abundant image contents include smooth movements [19, 20]. In this paper, prediction mode decision is regarded as the spatial characteristics of visual perception analysis. Consider

(6) S(x,y) ={2,mod⁡eP∈(Intra 16×16,Intra 4×4)1,(mod⁡eP∈Inter 8) or (mod⁡eI∈Intra 4×4)0.(mod⁡eP∈Inter 16) or (mod⁡eI∈Intra 16×16).

In formula (6), mod⁡ *e*_*P*_ represents the predicted mode of the current macroblock in frame *P*. mod⁡ *e*_*I*_ represents the predicted mode of the current macroblock in frame *I*.

If mod⁡ *e*_*P*_ chooses the intramode, *S*(*x*, *y*) = 2 means the spatial visual characteristic saliency is the highest and belongs to sensitive region.

If mod⁡ *e*_*P*_ chooses the inter 8 mode (inter 8 × 8, inter 8 × 4, inter 4 × 8, inter 4 × 4) or mod⁡ *e*_*I*_ chooses the intra 4 × 4 mode, *S*(*x*, *y*) = 1, means that the spatial visual characteristic saliency is high and belongs to attentive region.

If mod⁡ *e*_*P*_ chooses the inter 16 mode (skip, inter 16 × 16, inter 16 × 8, inter 8 × 16) or mod⁡ *e*_*I*_ chooses intra 16 × 16 mode, *S*(*x*, *y*) = 0, means that the spatial visual characteristic saliency is low and belongs to nonsaliency region.

## 3. Hierarchical Coding Scheme for VCL

H.264/AVC has higher compression, but the video coding complexity is increased continually, so it is a huge challenge to obtain the real-time performance. Some researches have shown that prediction mode decision and motion estimation (ME) occupy approximately 80% calculation in encoder [21]. Depending on the previous researches for fast mode decision algorithm and fast motion estimation algorithm, the computing resource will be optimized by intraprediction mode decision, interprediction mode decision, motion estimation search range, and numbers of references. The hierarchical video coding scheme proposed here is developed based on the visual perception characteristic analysis results according to the foregoing paragraphs.

### 3.1. Priority Setting for Visual Region of Interest

Based on the abundant video content and human visual selective attention principle, video sequences usually have temporal and spatial characteristics. The priority setting for visual region of interest is defined as

(7) $$\begin{array}{c} \text{ROI}\left(x,y\right)=\{\begin{array}{cc} 3, & \left(\left(M\left(x,y\right)=2\;\text{or}\;M\left(x,y\right)=1\right)||\left(S\left(x,y\right)=1\right)\right)\text{or}\left(S\left(x,y\right)=2\right)\\ 2, & \left(M\left(x,y\right)=2\;\text{or}\;M\left(x,y\right)=1\right)||\left(S\left(x,y\right)=0\right)\\ 1, & \left(M\left(x,y\right)=0\right)||\left(S\left(x,y\right)=1\right)\\ 0, & \left(M\left(x,y\right)=0\right)||\left(S\left(x,y\right)=0\right). \end{array} \end{array}$$

In formula (7), ROI(*x*, *y*) represents the priority setting for visual region of interest, *M*(*x*, *y*) represents the salient degree of temporal visual characteristic, *S*(*x*, *y*) represents the salient degree of spatial visual characteristic, and (*x*, *y*) represents the coordinates of the current macroblock.

### 3.2. Settings for the Resource Allocation Optimization

In order to improve the real-time performance while maintaining the video image quality and the compression bit rate, the macroblock with region of interest should be optimized firstly. With the limited computing resource and limited bits resource, the hierarchical video coding algorithm based on visual perception characteristics is proposed as shown in Table 1.

Fast intraprediction mode decision algorithm in Table 1 uses the macroblock histogram to define macroblock smoothness characteristics [19]. If the macroblock is flat, only Intra 16 × 16 mode is chosen. If the macro-block is rough, only Intra 4 × 4 mode is chosen. If the macroblock has nonsaliency texture, then Intra 16 × 16 mode and Intra 4 × 4 mode are ergodic.

Fast interprediction mode decision algorithm in Table 1 uses the early termination for some specified modes which are chosen by the probability of intermode decision [20].

Fast motion estimation search algorithm in Table 1 uses the dynamic search strategy which is proposed according to the correlation of motion vectors and the coding block motion degree [22].

(i) In Frame *P*, according to formula (7),.

**Case 1**

If the current macroblock belongs to foreground dynamic region (*M*(*x*, *y*) = 2) or foreground translational region (*M*(*x*, *y*) = 1), the macroblock has temporal visual characteristics. *S*(*x*, *y*) = 1 means that the macroblock chooses the inter 8 prediction mode and belongs to temporal visual characteristic saliency region.

**Case 2**

If *S*(*x*, *y*) = 2, frame *P* chooses the intraprediction mode and belongs to spatial visual characteristic saliency region.

Cases 1 and 2 include the highest human visual attention; the hierarchical video coding algorithm should perform the fast intraprediction mode, the inter 8 prediction mode, the fast motion estimation algorithm with Layer 2 to Layer 4, and 5 reference frames:  ROI(*x*, *y*) = 2.

**Case 1**

If the current macroblock has temporal visual characteristics (*M*(*x*, *y*) = 2or*M*(*x*, *y*) = 1), the macroblock has temporal visual characteristics but does not belong to saliency region. *S*(*x*, *y*) = 0 means that the macroblock only traverses the inter 16 prediction mode and skips the intra prediction mode.

This case includes the middle human visual attention; the hierarchical video coding algorithm should perform the inter 16 prediction mode and the fast motion estimation algorithm with Layer 1 to Layer 3, and 3 reference frames: ROI(*x*, *y*) = 1.

**Case 1**

If the current macroblock does not have temporal visual characteristics (*M*(*x*, *y*) = 0), the macroblock has spatial visual characteristics and belongs to spatial visual characteristic saliency region. *S*(*x*, *y*) = 0 means that the macroblock chooses the inter 8 prediction mode.

This case includes the lower human eye attention; the hierarchical video coding algorithm should perform the inter 8 prediction mode, the fast motion estimation algorithm with Layer 1 to Layer 2. and 2 reference frames: ROI(*x*, *y*) = 0.

**Case 1**

If the current macroblock does not have temporal and spatial visual characteristics, the macroblock belongs to background region.

This case includes the lowest human visual attention; the hierarchical video coding algorithm should perform the inter 16 prediction mode, the fast motion estimation algorithm with Layer 1, and one reference frame.

(ii) In Frame I, according to formula (7), ROI(*x*, *y*) = 1.

**Case 1**

If the current macroblock does not have temporal visual characteristics (*M*(*x*, *y*) = 0), the macroblock includes spatial details but does not belong to spatial visual characteristics saliency region. *S*(*x*, *y*) = 1 means that the macroblock chooses the intra 4 × 4 prediction mode.

This case includes the middle human visual attention; the hierarchical video coding algorithm should perform the intra 4 × 4 prediction mode: ROI(*x*, *y*) = 0.

**Case 1**

If the current macroblock does not have temporal and spatial visual characteristics, the macroblock belongs to stationary background region.

This case includes the lowest human visual attention; the hierarchical video coding algorithm should perform the intra 16 × 16 prediction mode.

## 4. Experimental Results and Analysis

In order to verify the rationality and the performance of the proposed hierarchical video coding algorithm, the experiment has been performed.

**Figure 4:**
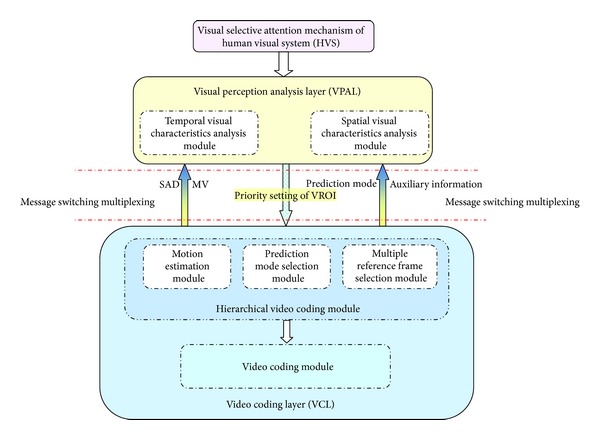
Video coding framework diagram.

The video coding framework diagram proposed in this paper is shown in Figure 4.

### 4.1. Environment and Configuration

(i) The standard video sequence: see Table 3.

(ii) System configuration: PC hardware configuration: Pentium 4, 2G RAM, 1.6 GHz frequency;

experimental software version: JM17.0, Visual C++ compiler, Windows 2003 operating system.

(iii) Main experimental parameter settings: Video sequence formats: QCIF; encoded frames: 100; frame rate: 30 f/s; GOP structure: IPPP; entropy coding type: CAVLC; QP: 28, 32, 36; motion estimation search range: ±16 pixels; the most number of reference frames: 5; Hadamard transform: On; rate-distortion optimization (RDO): On.

### 4.2. Experimental and Statistical Results

See Table 2.

### 4.3. Experimental Results and Performance Analysis

The statistic results in Table 2 show the performance of the hierarchical video coding scheme compared with the H.264/AVC (JM17.0) standard algorithm by ten typical sequences.

Compared with the H.264/AVC standard algorithm, under various QP (28, 32, and 36), the hierarchical video coding algorithm reduces 78.55%, 78.88%, and 79.22% coding time on average, the bit rate increases by 1.93%, 1.74%, and 1.27% on average (less than 3%), the PSNR-Y reduces 0.188 dB on average (the maximal reduce is less than 0.3 dB), in nonzero region with visual perception characteristics which is the human visual attention region, and the PSNR-Y reduces 0.153 dB on average (the maximal reduction is less than 0.25 dB). Compared with the human visual nonregion of interest, the hierarchical video coding scheme gives the priority to ensure the quality of the visual perception characteristics saliency region.

**Figure 5:**
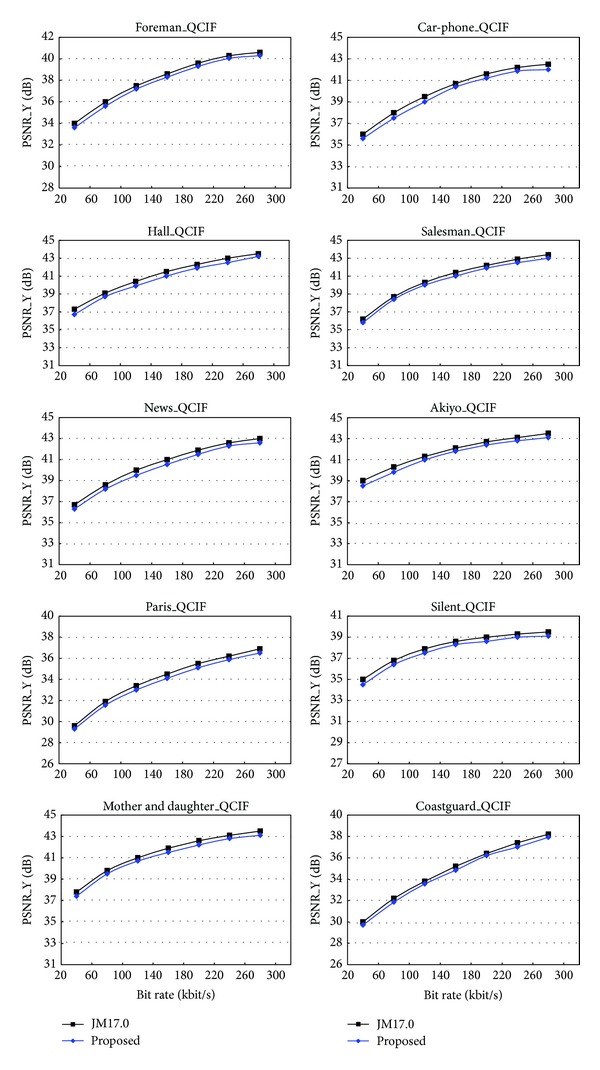
Comparison of the rate-distortion performance.

In terms of bit rate control, the two rate-distortion curves are very close as shown in Figure 5. It means that the proposed method inherits the advantages of low bit rate and high quality in H.264/AVC.

In terms of video image reconstruction quality, the proposed method ensures the average PSNR reduction to be less than 0.2 dB which is less than the perceived minimum human eyes sensitivity 0.5 dB. It maintains a good reconstructed video image quality.

**Figure 6:**
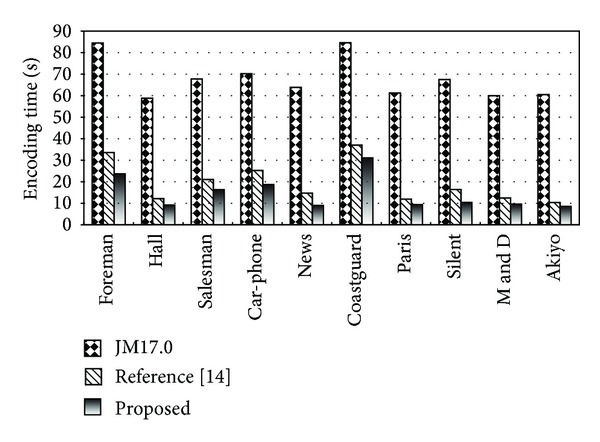
Comparison of the computational complexity

In terms of improving the coding computational speed, according to the statistical result as shown in Figure 6, the computational complexity of the proposed method is lower compared with the coding algorithm in reference [14] and H.264/AVC (JM17.0). It reduces about 85% coding time on average compared with the standard algorithm in H.264/AVC and fits for the sequences with gentle movements, such as Akiyo and News.

A large number of experimental results show that the proposed hierarchical video coding scheme based on visual perceptual analysis can accelerate the coding speed under the condition of maintaining good subjective video image quality. The experimental results also proved the feasibility of the low complexity visual perception analysis method based on the coding information. The consistency between visual perception characteristic saliency degree and HVS indicates the rationality of the hierarchical video coding algorithm based on visual perception characteristics.

## 5. Conclusion

This paper presents an efficient hierarchical video coding scheme based on visual perception characteristics. In order to achieve high coding performance, the scheme proposed video coding framework consisting of the video coding layer and the visual perception analysis layer. On one hand, the two layers can reduce the computation time greatly. The visual perception analysis layer uses the video stream information in coding layer to extract visual region of interest. On the other hand, the two layers can allocate the coding resource reasonably. The video coding layer uses the visual perceptive characteristics in perception analysis layer. The above technologies achieve a hierarchical video coding method and improve coding performance effectively. Experimental results show that the proposed algorithm can maintain good video image quality and coding efficiency; moreover, it can improve the H.264/AVC computational resource allocation. The proposed algorithm keeps the balance on good subjective video quality, high compression bit rate, and fast coding speed; also it lays the foundation for following the study of fast video coding algorithm in HEVC.

## Figures and Tables

**Table 1:** The hierarchical video coding algorithm based on visual perception characteristics.

Coding scheme	Intraprediction mode decision [19]		Interprediction mode decision [20]		ME search range [22]	Number of reference frames
	Intra 16 × 16	Intra 4 × 4	Inter 16	Inter 8		
Frame *P*						
ROI(*x*, *y*) = 3	Intra 16 × 16	Intra 4 × 4	—	Inter 8	Layers 2,3, 4	5
ROI(*x*, *y*) = 2	—	—	Inter 16	—	Layers 1, 2, 3	3
ROI(*x*, *y*) = 1	—	—	—	Inter 8	Layers 1, 2	2
ROI(*x*, *y*) = 0	—	—	Inter 16	—	Layer 1	1
Frame *I*						
ROI(*x*, *y*) = 1	—	Intra 4 × 4	—	—	—	—
ROI(*x*, *y*) = 0	Intra 16 × 16	—	—	—	—	—

“—”: no corresponding coding mode has been selected.

**Table 2:** Performance of the proposed algorithm compared with H.264/AVC standard.

Video sequence	QP	ΔTime (%)	ΔBit rate (%)	ΔPSNR-Y (dB)	ΔROI-PSNR-Y (dB)
Foreman	28	−71.24	+2.19	−0.30	−0.25
	32	−71.92	+2.15	−0.29	−0.21
	36	−70.81	+2.06	−0.17	−0.15

Hall	28	−83.64	+1.24	−0.21	−0.19
	32	−84.27	+1.17	−0.22	−0.14
	36	−84.98	+1.12	−0.19	−0.13

Salesman	28	−76.43	+2.12	−0.26	−0.21
	32	−75.92	+1.87	−0.20	−0.15
	36	−76.01	+1.83	−0.14	−0.12

Car-phone	28	−73.85	+2.84	−0.32	−0.24
	32	−73.36	+2.57	−0.21	−0.19
	36	−74.96	+1.69	−0.14	−0.12

News	28	−85.47	+2.14	−0.13	−0.11
	32	−85.84	+2.13	−0.12	−0.07
	36	−85.93	+1.86	−0.09	−0.08

Coastguard	28	−61.24	+2.47	−0.28	−0.24
	32	−62.35	+2.13	−0.29	−0.26
	36	−62.62	+1.76	−0.21	−0.18

Paris	28	−84.12	+1.07	−0.29	−0.23
	32	−84.61	+1.15	−0.22	−0.21
	36	−84.97	+1.03	−0.21	−0.17

Silent	28	−80.14	+1.51	−0.24	−0.18
	32	−80.86	+1.42	−0.18	−0.15
	36	−81.32	+1.17	−0.11	−0.09

Mother and Daughter	28	−83.76	+1.85	−0.18	−0.14
	32	−83.89	+1.64	−0.10	−0.10
	36	−84.21	+0.09	−0.08	−0.05

Akiyo	28	−85.64	+1.87	−0.11	−0.09
	32	−85.76	+1.21	−0.08	−0.07
	36	−86.41	+0.08	−0.07	−0.06

Average results					
QP	28	−78.55	+1.93	−0.188	−0.153
	32	−78.88	+1.74		
	36	−79.22	+1.27		

Description: The symbol “+” means enhancement or increase; symbol “−” means decrement or decrease. PSNR-Y means the peak signal-to-noise ratio of luminance, and it also represents the quality of the reconstructed video image. ΔPSNR-Y means the difference of the PSNR-Y. ΔROI-PSNR-Y means the non-zero region of the ΔPSNR-Y in visual perception characteristics mark.

**Table 3:** The standard video sequence.